# A P-Loop NTPase Regulates Quiescent Center Cell Division and Distal Stem Cell Identity through the Regulation of ROS Homeostasis in *Arabidopsis* Root

**DOI:** 10.1371/journal.pgen.1006175

**Published:** 2016-09-01

**Authors:** Qianqian Yu, Huiyu Tian, Kun Yue, Jiajia Liu, Bing Zhang, Xugang Li, Zhaojun Ding

**Affiliations:** 1 The Key Laboratory of Plant Cell Engineering and Germplasm Innovation, Ministry of Education, College of Life Science, Shandong University, Jinan, People’s Republic of China; 2 School of Biological Science and Technology, University of Jinan, Jinan, People’s Republic of China; Duke, UNITED STATES

## Abstract

Reactive oxygen species (ROS) are recognized as important regulators of cell division and differentiation. The *Arabidopsis thaliana* P-loop NTPase encoded by *APP1* affects root stem cell niche identity through its control of local ROS homeostasis. The disruption of APP1 is accompanied by a reduction in ROS level, a rise in the rate of cell division in the quiescent center (QC) and the promotion of root distal stem cell (DSC) differentiation. Both the higher level of ROS induced in the *app1* mutant by exposure to methyl viologen (MV), and treatment with hydrogen peroxide (H_2_O_2_) rescued the mutant phenotype, implying that both the increased rate of cell division in the QC and the enhancement in root DSC differentiation can be attributed to a low level of ROS. *APP1* is expressed in the root apical meristem cell mitochondria, and its product is associated with ATP hydrolase activity. The key transcription factors, which are defining root distal stem niche, such as *SCARECROW* (*SCR*) and *SHORT ROOT* (*SHR*) are both significantly down-regulated at both the transcriptional and protein level in the *app1* mutant, indicating that *SHR* and *SCR* are important downstream targets of APP1-regulated ROS signaling to control the identity of root QC and DSCs.

## Author Summary

Reactive oxygen species (ROS) are recognized as important regulators of cell division and differentiation. In this study, we characterized an *Arabidopsis thaliana* P-loop NTPase encoded by *APP1* regulates root stem cell niche identity through its control of local ROS homeostasis. The *app1* mutant shows a reduction in ROS level, a rise in the rate of cell division in the quiescent center (QC) and the promotion of root distal stem cell (DSC) differentiation. The increased rate of cell division in the QC and the enhancement in root DSC differentiation in *app1* can be attributed to a low level of ROS since both the exposure to methyl viologen (MV), and treatment with hydrogen peroxide (H_2_O_2_) rescued the mutant phenotype. *APP1* is expressed in the root apical meristem cell mitochondria, and its product is associated with ATP hydrolase activity. The key transcription factors such as *SCARECROW* (*SCR*) and *SHORT ROOT* (*SHR*), which are defining root distal stem niche, are both greatly down-regulated at both the transcriptional and protein level in *app1*, indicating that *SHR* and *SCR* are important downstream targets of APP1-regulated ROS signaling to control the identity of root QC and DSCs.

## Introduction

Root growth is maintained by the root apical meristem, which harbors the so-called “quiescent center” (QC) and its surrounding stem cells within the stem cell niche (SCN) [1]. In the *Arabidopsis thaliana* root, the QC comprises a small group of cells which divide infrequently [2]. The structure ensures that the surrounding stem cells divide asymmetrically, thereby avoiding terminal differentiation [3,4].

In *Arabidopsis*, the root apical meristem (RAM) has multiple molecular signal modules that regulate SCN. The GRAS transcription factor SCARECROW (SCR) is required to establish the QC identity and root stem cell activity [5]. SCR sustains root stem cell and root meristem activity by suppressing cytokinin response transcription factors ARABIDOPSIS RESPONSE REGULATOR 1 (ARR1) and ARABIDOPSIS RESPONSE REGULATOR 12 (ARR12) [6,7]. In addition, the transcription factor MYB DOMAIN PROTEIN 36 (MYB36), which regulates the transition from proliferation to differentiation in the endodermis, was recently identified to be directly activated by SCR [8]. SCR was also found to control the QC identity through the direct interaction with RETINOBLASTOMA-RELATED (RBR) protein, which acts in a cell-autonomous manner to maintain the quiescence of the QC [9]. *SCR* is regulated by another GRAS transcription factor SHORT ROOT (SHR), which is expressed in the stele and moves into the surrounding tissue layer to directly activate *SCR* expression by binding to the *SCR* promoter. SCR forms a heterodimer with SHR to inhibit the binding of SHR at the *SCR* promoter [6,10]. In parallel, PLETHORA (PLT) AP2-domain transcription factors are essential for QC specification and stem cell activity in an auxin dependent manner [11]. Distal *PLT* transcript accumulation is overlapped with the radial expression domains of *SCR* and *SHR*, providing positional information for the root SCN [11].

The WUSCHEL-RELATED HOMEOBOX 5 (WOX5) homeodomain transcription factor is specifically expressed in the QC to regulate QC and root distal stem cell (DSC) identity, and the *wox5* mutant exhibits the absence of root DSCs [12,13,14]. Recently, it was found that the WOX5 protein moves from the root QC into the root DSCs, where it directly represses the differentiation factor CYCLING DOF FACTOR 4 (CDF4) to maintain the root stem cell identity [15]. In addition, the WOX5 regulated root stem cell identity was realized through the suppression of cell cycle related genes such as CYCD3;3 and CYCD1;1 in the QC [15,16]. On the other hand, the expression of *WOX5* was found to be regulated via ARF10 and ARF16 auxin response factors and was also under the control of proximal meristem expressed REPRESSOR OF WUSCHEL1 (ROW1) [17,18].

The ATP-dependent SWI/SNF chromatin remodeling complexes, which regulate gene transcription by using the energy of ATP hydrolysis, have been shown to play a critical role in animal development and cell differentiation [19,20]. In *Arabidopsis*, BRAHMA (BRM), a SWI/SNF chromatin remodeling ATPase, was reported to control root SCN maintenance through directly targeting to the chromatin of auxin efflux carrier encoding genes such as *PIN1*, *PIN2*, *PIN3*, *PIN4*, and *PIN7* and regulating auxin gradient in root tips [21]. In addition, the elongator complex subunit 2 (ELP2) protein, one subunit of an evolutionarily conserved histone acetyltransferase complex, was also reported to regulate the expression and polarity of auxin efflux carrier *PIN1* and *PIN2*, thus caused reduced auxin accumulations in root tips and root development [22]. Furthermore, the defected root development in *elp2* was also attributed to the down-regulated expression of root stem cell defining factors such as PLT1, PLT2, SHR and SCR through ELP2 mediated epigenetic modifications [22].

Reactive oxygen species (ROS) were initially deemed to be toxic by-products, but are now recognized as acting as secondary messengers regulating cell growth and differentiation [23,24]. ROS is important for the control of cell division and cell differentiation, and it is involved into the maintenance of the continuing renewal and differentiation of animal stem cells, which are sensitive to ROS [25]. ROS gradients formed in the primary root are important for controlling the transition between root cell proliferation and differentiation, and thus control root growth [24]. Abscisic acid (ABA)-promoted ROS production in the root tip cell mitochondria acts as a retrograde signal to regulate root meristem activity by controlling *PLTs* transcription [26]. However, the role of ROS in the maintenance of QC and root DSC identity has not so far been explored in any detail. Here, the P-loop NTPase *APP1* has been shown to regulate QC and root DSC identity via the regulation of ROS homeostasis. The NTPases, which hydrolyze nucleoside triphosphates, are important for signal transduction, cell division, transcription and translation. *APP1* was shown to display ATPase activity *in vitro* and to modulate the level of ROS in the *A*. *thaliana* root. These findings provide a direct link between ROS distribution and the QC identity and root DSC differentiation in the *A*. *thaliana* root tip.

## Results

### APP1 affects the QC and DSC identity in *Arabidopsis* root

**Fig 1:**
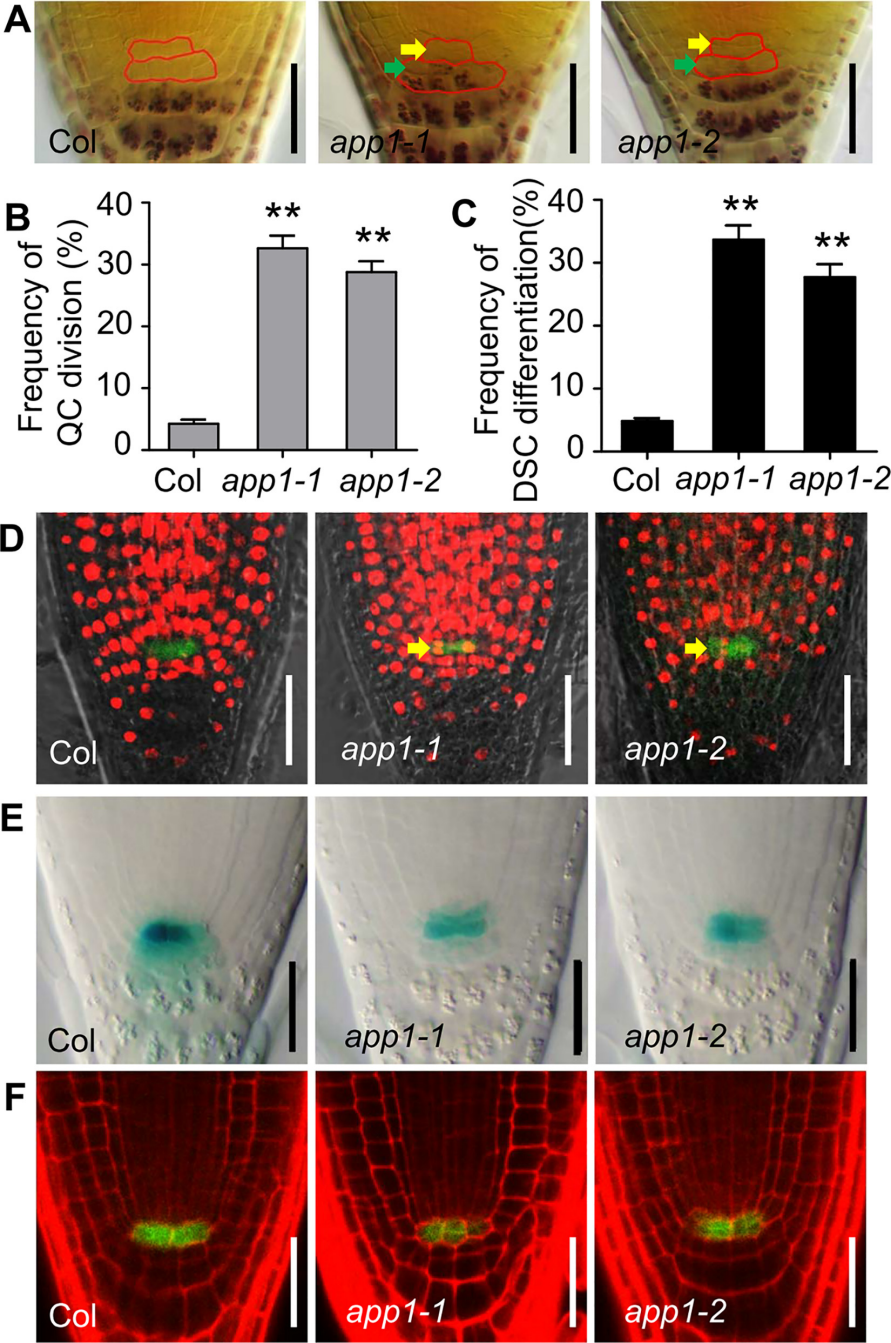
Root SCN maintenance is defective in the absence of functional APP1. (A) In Lugol-stained five-day-old *app1* mutant roots, cell division in the QC is enhanced (yellow arrowheads show cell division in the irregular QC cells) and root DSC differentiation is encouraged (green arrowheads show starch accumulation in the irregular DSCs). (B, C) Quantitative evaluation of QC cell division and DSC differentiation in Col and the *app1* mutant roots. At least 100 roots were examined per genotype per experiment. Error bars represent SE from triplicate experiments. **P < 0.01, Student’s t-test. (D) Confocal micrography illustrating the incorporation of EdU into WT and the *app1* mutant QC cells. Red fluorescence of EdU-positive nuclei in QC cells of the *app1* mutant roots indicates that QC is in a state of active division (yellow arrow). Green fluorescence of *pWOX5*::*GFP* mark the QC cells. (E) GUS staining of the roots of transgenic plants (either WT or the *app1* mutant) harboring *QC184* shows that reduced expression of *QC184* in the *app1* mutants. (F) Mutation of *APP1* induced QC cell division revealed by the QC marker *pWOX5*::*GFP*. Scale bars in (A, D-F): 50 μm.

To identify new regulators that are involved in root stem cell maintenance, a T-DNA mutant screening was done using Lugol’s staining, which normally stains only differentiated starch-containing columella cells, and leads to the identification of the *app1-1* mutant that displayed aberrant QC cell divisions along with an inability to maintain its DSCs (Fig 1). In 5-day-old wild type (WT), most of the roots (>95%) had only one layer of DSC, as demonstrated by the absence of the Lugol’s staining. However, the 5-day-old *app1-1* mutant roots displayed higher rates (around 30%) of DSC differentiation which was shown by the starch granule accumulation (Fig 1A and 1C). In the WT, the QC cells divide only infrequently (Fig 1A and 1B). In contrast, in the *app1-1* mutant, a higher frequency of QC cell division (>20%, compared to the WT rate of 5%) was observed (Fig 1A and 1B). When 5-day-old seedlings were exposed to EdU (an S phase progression fluorescence dye), a clear red fluorescent signal was observed in the QC cells (marked with *pWOX5*::*GFP*) of the *app1-1* mutants roots, while it was absent in the WT control (Fig 1D). Using the QC-specific markers such as *QC184* and *pWOX5*::*GFP* (for green fluorescent protein), the dividing QC cells were clearly observed (Fig 1E and 1F). Though *pWOX5*::*GFP* expression was not altered in the *app1* mutants (Fig 1F and S2D Fig), the expression of the QC-specific reporter *QC184*::*GUS* was clearly down-regulated (Fig 1E). Thus both the QC identity and the balance of cell proliferation and differentiation in the root DSC were disrupted in the *app1-1* mutants.

A Tail-PCR analysis identified that the *app1-1* (*At*5*g*53540) allele was interrupted by the insertion of a T-DNA element within the gene's first intron (S1A Fig). This mutation was confirmed through analysis of a second *app1* mutant allele (*app1-2*, *Salk_091643*). The *app1-2* mutant and the *app1-1 app1-2* F_1_ hybrid shared the same phenotype as the *app1-1* mutant (Fig 1 and S1B Fig). In both mutants, the abundance of *APP1* transcript was greatly reduced (S1C Fig). *APP1* promoter driving *APP1* cDNA fused with *GFP* in frame (*pAPP1*:*APP1-GFP*) complemented the QC cell division and root DSC differentiation phenotype in the *app1-1* and *app1-2* mutants (S1D Fig). These results confirmed that the mutations in *APP1* led to the phenotypes of the *app1* mutants. In addition, both the *app1* mutants and the *APP1* over-expression lines produced roots of normal length, suggesting the specific role of *APP1* in the maintenance of root QC and DSC identity (S2A–S2C Fig).

### APP1 is highly expressed in root meristem and localized to mitochondria

**Fig 2:**
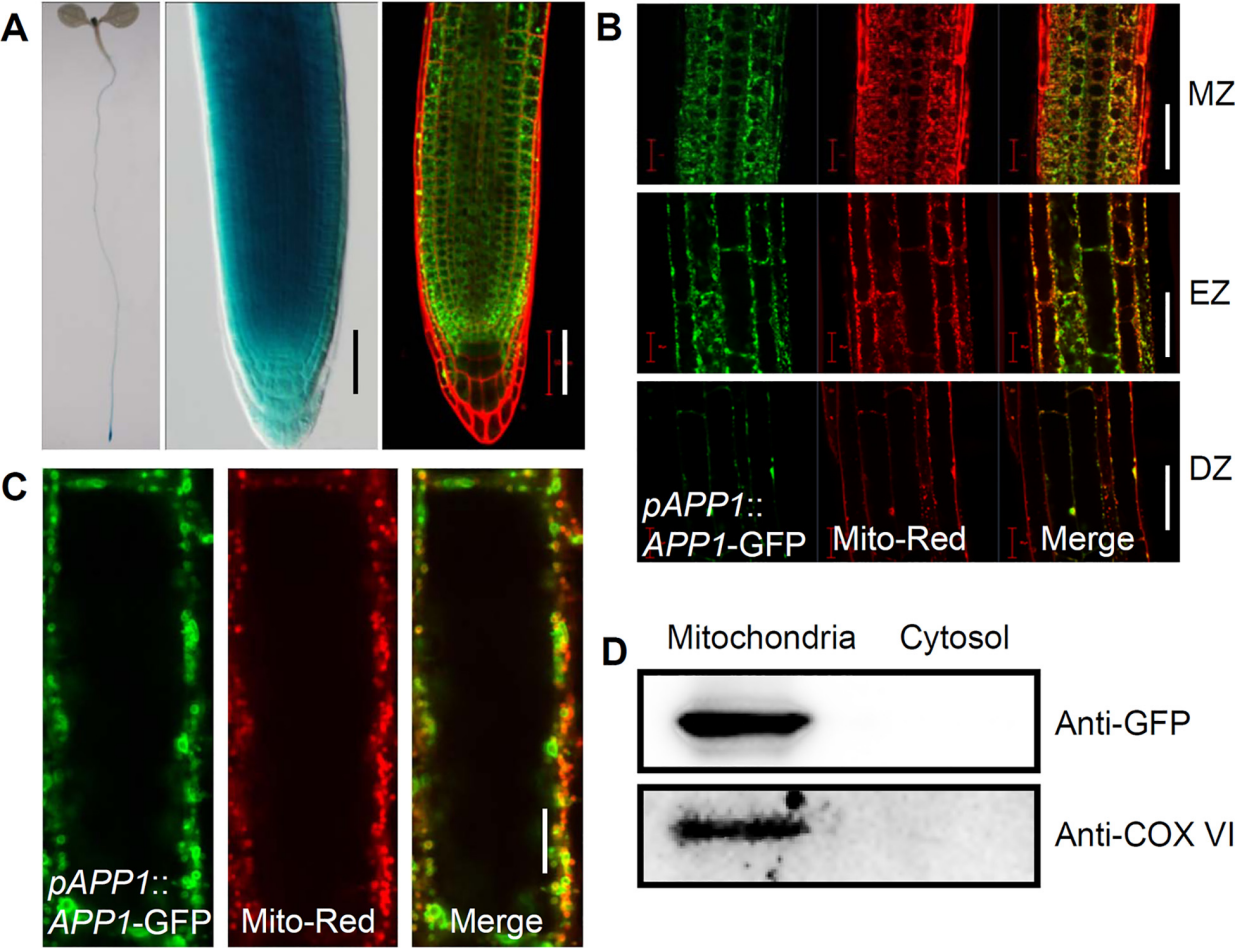
The expression profile and sub-cellular localization of APP1. (A) GUS staining analysis of the *pAPP1*::*GUS* transgenic seedlings. Confocal image of *pAPP1*::*APP1-GFP* in the root tip (GFP, green; PI, red). Scale bar: 50 μm. (B-C) The *p*APP1::APP1-GFP (GFP, green) is co-localized with the mitochondria marker, Mito-Tracker (red), in MZ, EZ, DZ (B) and single cell level (C). MZ, meristem zone; EZ, elongation zone; DZ, differentiation zone. (D) APP1-GFP were localized to mitochondria which was shown by western blotting analysis with proteins isolated from *pAPP1*::*APP1-GFP* seedlings. Mitochondria marker protein COX IV was used as a positive control for this analysis. Scale bars: 50 μm (B), 10 μm (C).

To assess the possible role of *APP1* in plant growth, we generated *pAPP1*::*GUS-GFP* (*APP1* promoter fused with *GUS-GFP*) and *pAPP1*::*APP1-GFP* (*APP1* promoter driving *APP1* cDNA fused with *GFP* in frame) lines to study the *APP1* expression pattern and the APP1 protein sub-cellular localization. Analysis of several *pAPP1*::*GUS-GFP* and *pAPP1*::*APP1-GFP* transgenic lines revealed that the GUS activity or GFP signals were highly detected in the root meristem, and also slightly expressed in shoot meristem and young leaves (Fig 2A). Expression patterns were similar for both *pAPP1*::*APP1-GFP* and *pAPP1*::*GUS-GFP* transgenic lines. Further study showed that APP1-GFP green fluorescence mainly merged with the red fluorescence of the mitochondrial probe, Mito Tracker Red (Fig 2B and 2C), indicating a main mitochondria localization of APP1. This result was also confirmed by western blotting analysis with the isolation of mitochondria proteins from *pAPP1*::*APP1-GFP* seedlings. The result showed that both APP1-GFP and mitochondrial marker protein COX IV (cytochrome c oxidase complex IV) were detected in the mitochondria proteins instead of other components in cytosol (Fig 2D).

### The loss of *APP1* lowers the concentration of superoxide in the root tip

**Fig 3:**
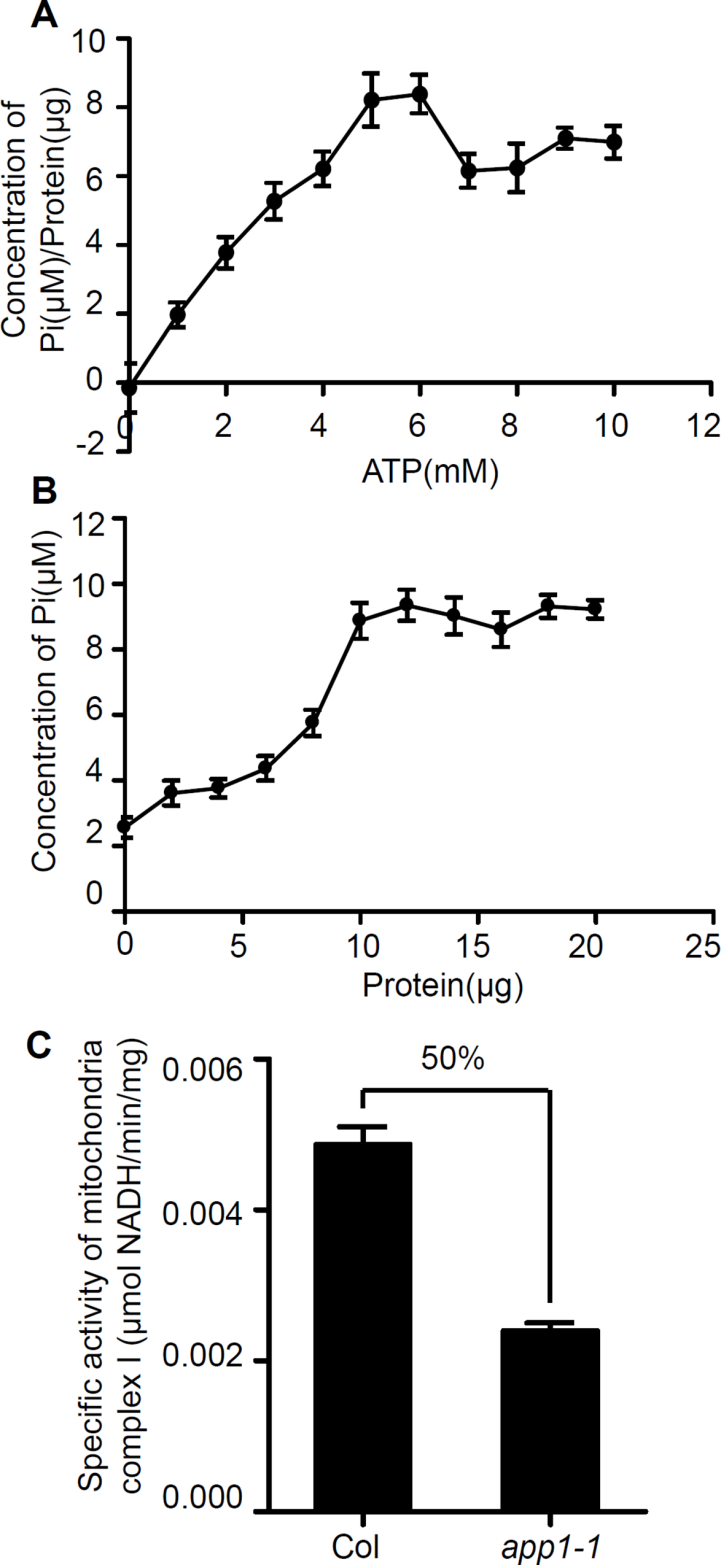
The *in vitro* ATP hydrolysis assay of APP1 protein. (A) The rate of ATP hydrolysis as a function of ATP concentration. Hydrolysis was most effective when the reaction was provided with 5 mM ATP. (B) The effect of APP1 dosage on ATP hydrolysis, increasing the amount of APP1 present (up to 10 μg) had the effect of increasing the quantity of free Pi generated; above this level, the reaction appeared to be saturated. (C) The activity of mitochondrial complex I was reduced by 50% in the *app1* mutant. Error bars represent SE from triplicate experiments.

**Fig 4:**
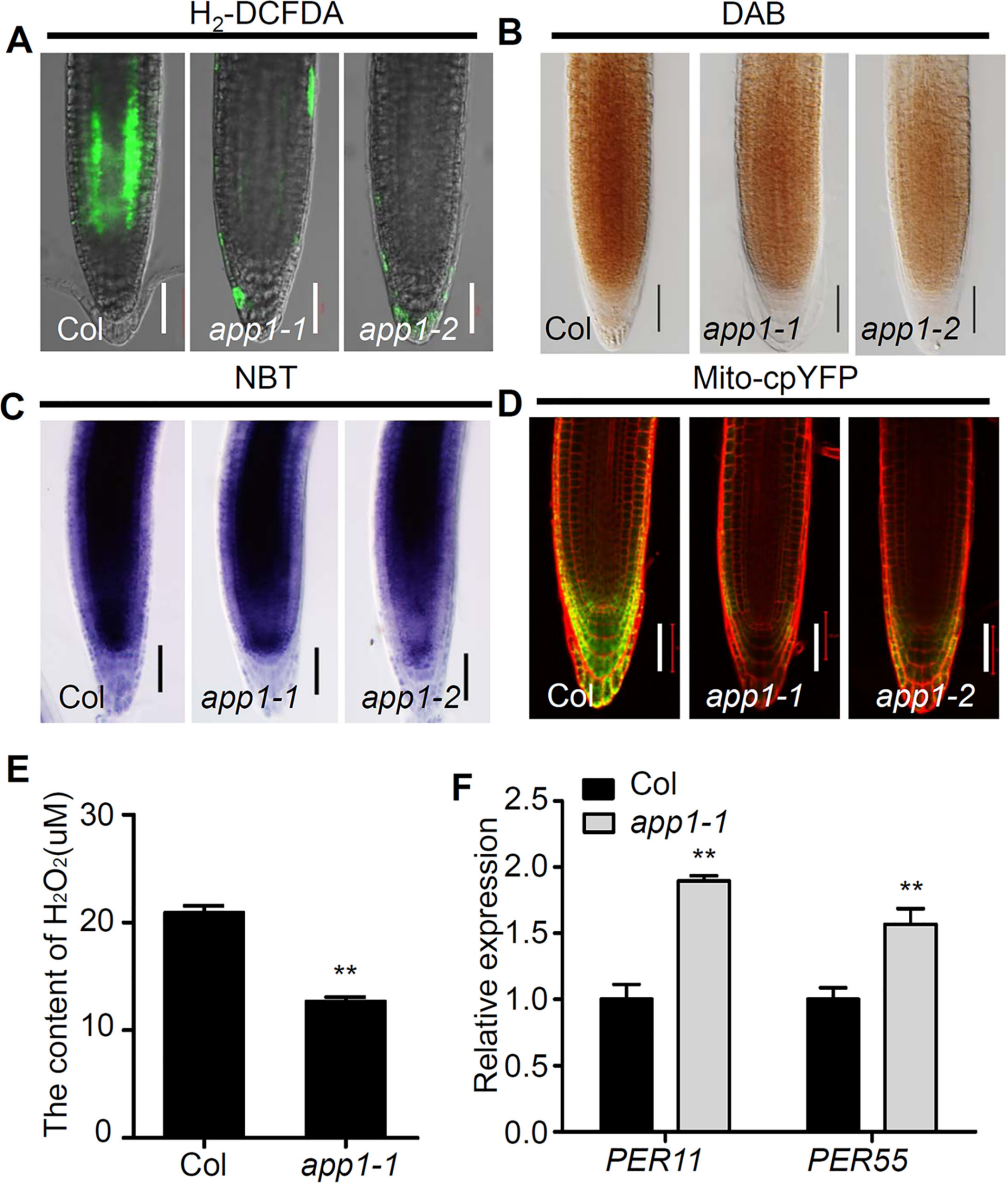
The *app1* mutants accumulate less ROS than the WT. (A) H2-DCFDA staining for ROS in primary root tips of WT and the *app1* mutants. (B) DAB staining for H_2_O_2_ in primary root tips of Col and the *app1* mutants. (C) NBT staining for O^2-^ in primary root tips of WT and the *app1* mutants. (D) Fluorescence analysis of Mitocp-YFP in Col and the *app1* mutant roots. (E) The H_2_O_2_ level in the *app1* mutant was significantly reduced compared to Col. (F) The expression level of *PER11* and *PER55* were increased in the *app1* mutant which is revealed by qRT-PCR. The data are given in the form of the mean with an associated SD (*n* = 3); **: P<0.01, Student’s-t test. Bars in (A-D): 50 μm.

Since APP1 resembles a P-loop AAA type ATPase protein, an *in vitro* ATP hydrolysis assay was performed. His fusion APP1 protein was isolated from E. coli. Hydrolysis was most effective when the reaction was provided with 5 mM ATP (Fig 3A). At a fixed level of ATP (2mM), increasing the amount of APP1 protein present (up to 10 μg) could increase the generation of free Pi quantity, and above this level, the reaction appeared to be saturated (Fig 3B). An inspection of the mitochondrial complex I protein showed that its specific activity in the *app1-1* mutant was only 50% of that in the WT (Fig 3C). Since the mitochondria represent the main source of ROS in the plant cell, and the hydrolysis of ATP increases the proton motive force, thereby enhancing ROS production [27], we next examined if the affected mitochondria activity causes ROS changes in the *app1* mutants. Compared to WT root tips, those of the *app1* mutant seedlings accumulated substantially less hydrogen peroxide (indicated by DAB and H2-DCFDA staining) and superoxide (using NBT staining) (Fig 4A–4C). When the mitochondrial superoxide marker *Mito-cpYFP* [28] was used to monitor ROS accumulation, the signal was also greatly reduced in the *app1* mutants compared to the WT (Fig 4D). In addition, the level of hydrogen peroxide (H_2_O_2_) was lower in the *app1* mutant compared to the WT control (Fig 4E). These results suggest that dysfunction of *APP1* reduces ROS production in root tips, as supported by the up-regulation of *PER11* and *PER55*, which both encode ROS-scavenging enzymes and belong to the Class III peroxidase family [29] (Fig 4F).

### Altered ROS levels induce an increased rate of QC cell division and root DSC differentiation

**Fig 5:**
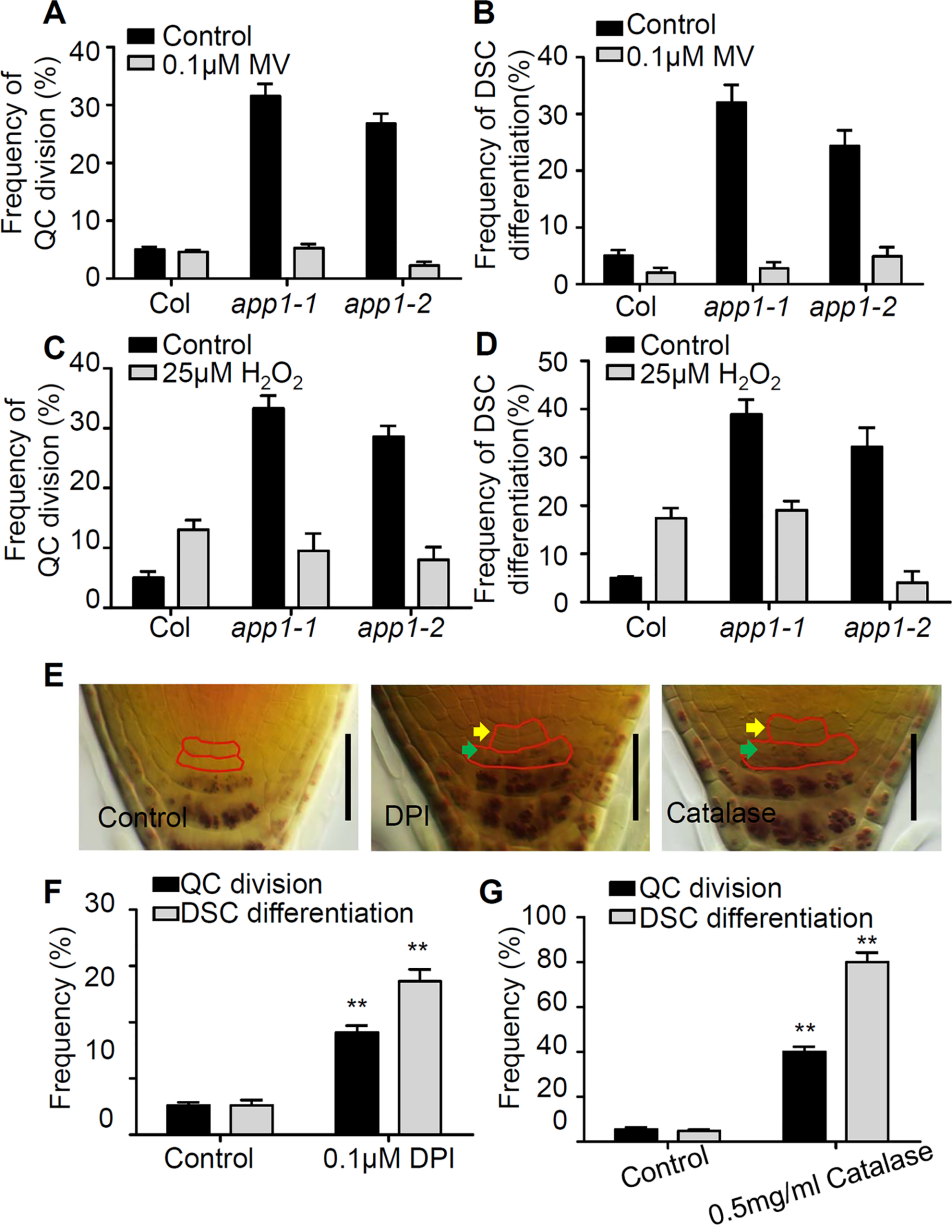
A low ROS level promotes QC cell division and root DSC differentiation. Quantification of (A) QC cell division and (B) root DSC differentiation in the absence (black bar) or the presence (gray bar) of 0.1 μM MV. Quantification of (C) QC cell division and (D) root DSC differentiation in the absence (black bar) or presence (gray bar) of 25 μM H_2_O_2_. (E) Lugol staining of five-day-old roots without or with DPI or catalase treatment. QC cells and DSC were labeled with red solid lines (yellow arrows show divided QC cells, green arrows show differentiated DSC cells). Bar: 50 μm. (F, G) Quantification of QC cell division (black bar) and root DSC differentiation (gray bar) in roots exposed to 0.1 μM DPI or 0.5 mg/ml catalase. At least 100 seedlings were examined for each time point for each biological repeat. Error bars represent SE from triplicate experiments. **: P<0.01, Student’s t-test.

To test if the increased rate of QC cell division and the enhanced root DSC differentiation were attributed to the reduced ROS levels in *app1* mutants, the effect on the root SCN of both providing hydrogen peroxide (H_2_O_2_) exogenously or exposing the plant to methyl viologen (MV) (which causes an overproduction superoxide, O^2-^) was examined. Both 25 μM H_2_O_2_ and 0.1 μM MV treatments strongly rescued the *app1* mutant phenotype in root SCN (Fig 5A–5D). This rescued *app1* mutant phenotype in root SCN might be result from the increased ROS level by the treatment of H_2_O_2_ and MV which both increase ROS levels shown by using H_2_-DCFDA staining and the mitochondrial superoxide marker *Mito-cpYFP* (S3 Fig). When WT seedlings were grown on a medium supplemented with either diphenyleneiodonium (DPI) (a reagent which inhibits both NOX-dependent ROS production and ROS production in the mitochondria) or catalase (which converts hydrogen peroxide into water) [30, 31], both treatments significantly induced QC cell division and boosted root DSC differentiation by reducing ROS levels (Fig 5E–5G, S3 Fig).

**Fig 6:**
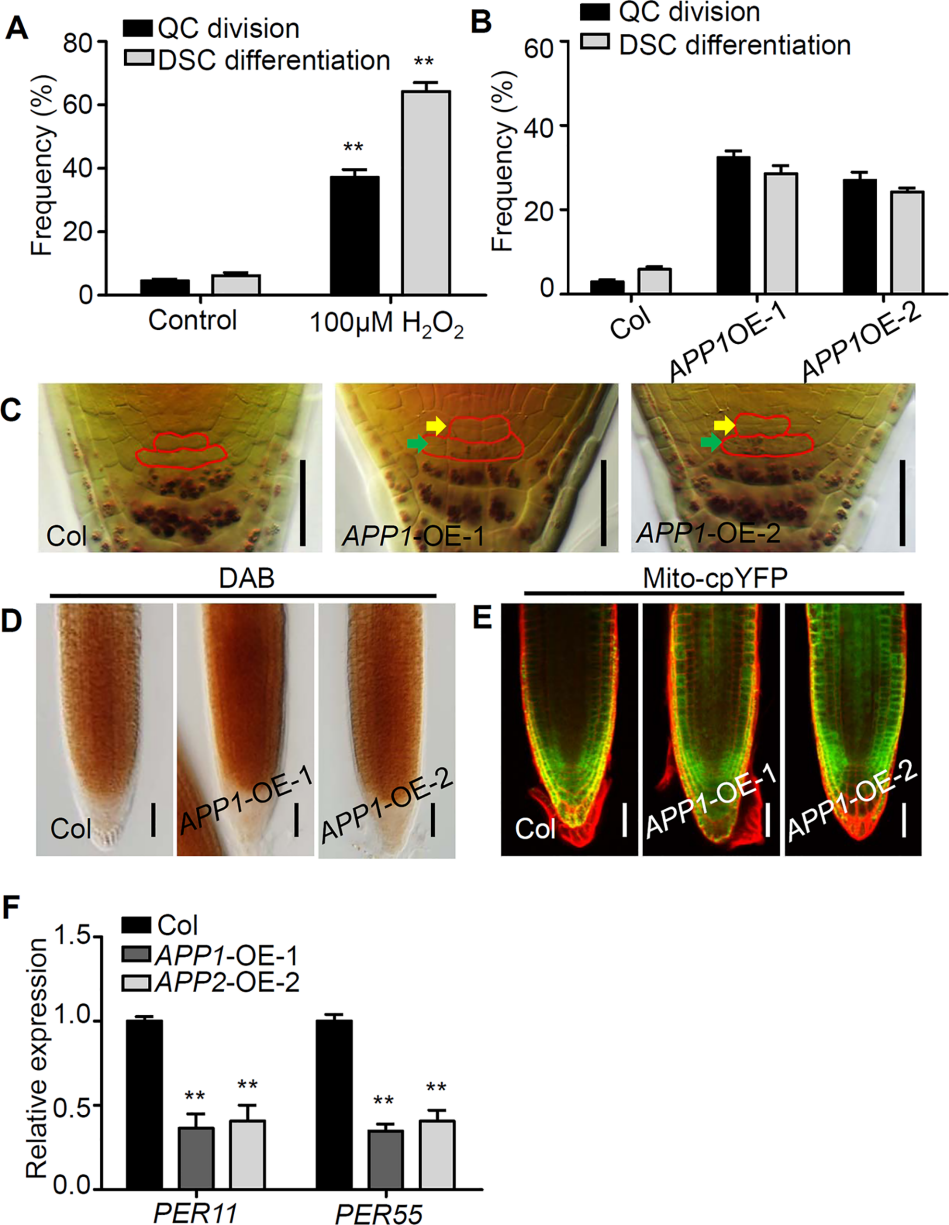
The elevated ROS level in the *APP1* over-expression line promotes QC cell division and root DSC differentiation. (A) Quantification of QC cell division (black bar) and root DSC differentiation (gray bar) in roots exposed to 100μM H_2_O_2_. (B) Quantification of QC cell division (black bar) and root DSC differentiation (gray bar) in the roots of WT and the *APP1* over-expression lines. (C) Lugol-stained five-day-old roots of WT and the *APP1* over-expression lines. QC cells and DSC were labeled with red solid lines (yellow arrow show divided QC cells, green arrow show differentiated DSC cells). (D) DAB staining for H_2_O_2_ in primary root tips of Col and *APP1-OE* lines. (E) Fluorescence analysis of Mitocp-YFP in Col and *APP1-OE* roots. (F) The expression levels of *PER11* and *PER55* were reduced in the *APP1-OE* lines which were revealed by qRT-PCR. At least 50 seedlings were examined for each time point for each biological repeat. Error bars represent SE from triplicate experiments. **: P<0.01, Student’s t-test. Scale bars in (C, D, E): 50 μm.

When WT seedlings were exposed to 25 μM H_2_O_2_, the rate of cell division in the QC was increased by around 15% and the extent of root DSC differentiation promoted by around 20% (Fig 5C and 5D). Increasing the concentration of H_2_O_2_ to 100 μM induced an even stronger phenotype, with around 40% of seedlings displaying QC cell division and more than 60% exhibiting root DSC differentiation (Fig 6A). Consistently, in the *APP1* over-expression lines, which were engineered by introducing the construct 35S::*APP1* and displayed higher ROS levels indicated by the DAB staining and H_2_-DCFDA staining (Fig 6D and 6E, S1E Fig), the QC cell division rate was boosted to around 30% and the rate of root DSC differentiation was enhanced to 20–30% (Fig 6B and 6C). Consistently, the ROS-scavenging enzyme encoding genes such as PER11 and PER55 were down-regulated at the transcriptional level in *APP1* over-expression lines (Fig 6F). In addition, we also expressed *APP1* in the QC using the *WOX5* promoter in WT *Arabidopsis* root, and the wild-type transgenic seedlings displayed increased QC cell division and enhanced root DSC differentiation, while in both *app1* mutant background, the *pWOX5*::*APP1* could only partially repress the aberrant QC cell division and root DSC differentiation (S4 Fig). The conclusion was that *APP1* functions as a regulator of ROS production, which in turn governs cell division in the QC and the maintenance of root DSC identity.

### *SCR* and *SHR* are down-regulated in the *app1* mutant

**Fig 7:**
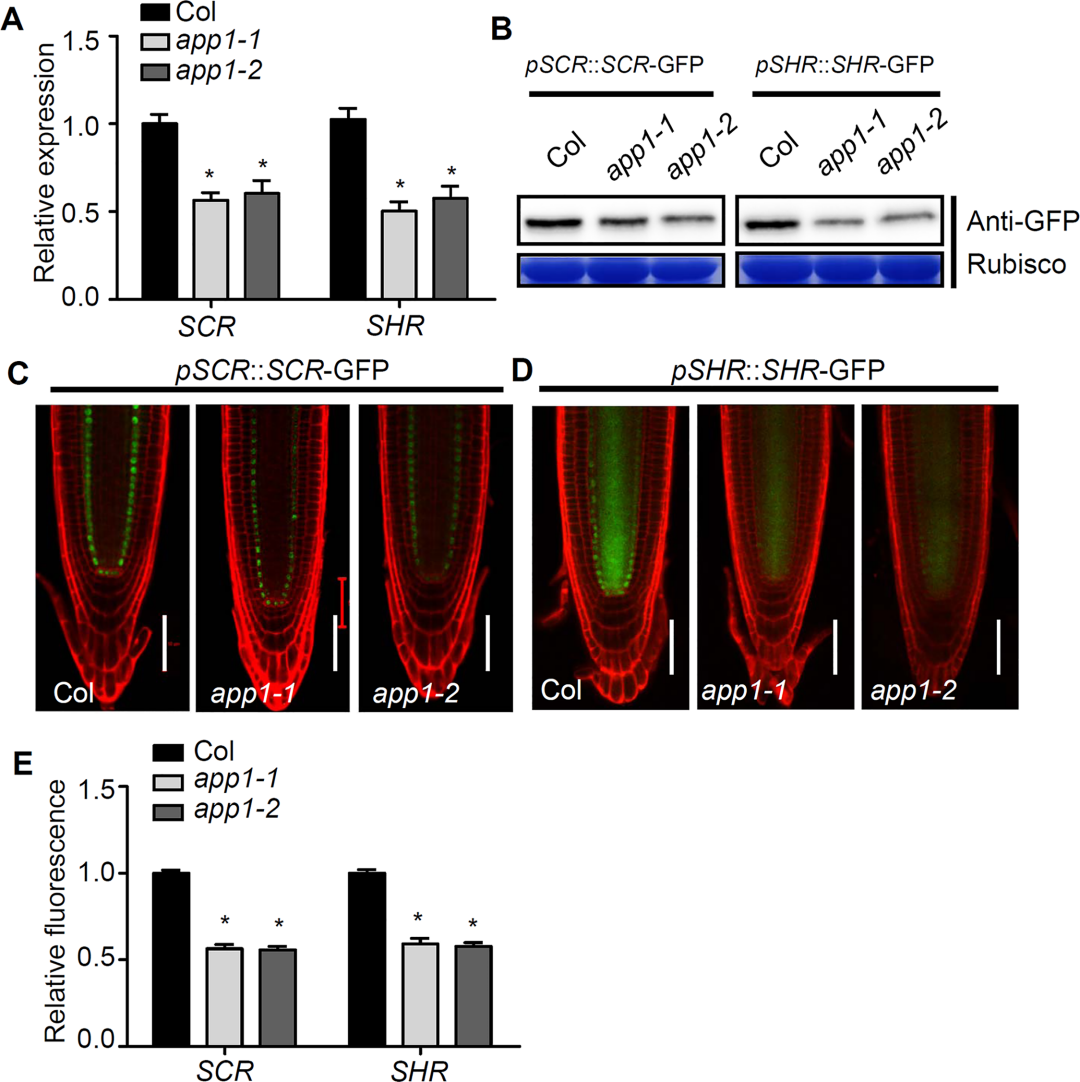
*SCR* and *SHR* expression are reduced in the absence of *APP1*. (A) The reduced expression of *SCR* and *SHR* in *app1* was confirmed by qRT-PCR analysis. (B) Immunoblotting analysis of SCR in *pSCR*::*SCR*-*GFP*/Col and *pSCR*::*SCR-GFP/app1* transgenes and SHR in *pSHR*::*SHR*-*GFP*/Col and *pSHR*::*SHR-GFP/app1* transgenes. Loading controls in immunoblotting experiments were Rubisco stained with Coomassie Brilliant Blue. (C, D)Expression of the transgenes *pSCR*::*SCR-GFP* and *pSHR*::*SHR-GFP* were reduced in the *app1* mutants. Bar: 50 μm. (E) Quantification of GFP fluorescence shown in (C, D). At least 50 seedlings were examined for each time point for each biological repeat. The data are given in the form of the mean with an associated SD (*n* = 3); *, P<0.05, Student’s t test.

To determine whether APP1 regulated root SCN is dependent on the well characterized root SCN-defining transcription factors, we examined the expression of the HOMEOBOX 5 transcription factor gene *WOX5*, AP2 transcription factor genes such as *PLT1* and *PLT2* and GRAS transcription factor genes such as *SCR* and *SHR* in *app1*. The expression of *SHR* and *SCR* were transcriptionally down-regulated in *app1* which were shown by qRT-PCR analysis (Fig 7A). Consistently, the protein expression levels of both SHR and SCR were also greatly reduced which were shown by western blot analysis or the pSCR::SCR-GFP and pSHR::SHR-GFP fluorescence examinations in *app1* (Fig 7B–7E). Other transcription factor genes such as *WOX5*, *PLT1*, *PLT2* and cell cycle gene *CYCB1* displayed the similar expression levels in the *app1* mutant roots compared to the WT control (S5 Fig). Accordingly, the expression levels of *PLT1* and *PLT2* were not affected in APP1-OE lines (S6 Fig). In addition, auxin signaling, which has been known to play a crucial role in root SCN maintenance, was not altered in *app1* which was shown by the similar DR5rev::GFP signals between WT and the *app1* mutant root tips (S7 Fig). Furthermore, the expression of *UPBEAT1* (*UPB1*), encoding a bHLH transcription factor which regulates root meristem through the modulation of the ROS gradient in root (24), was not changed in *app1* and the *APP1* expression also showed wild-type pattern in *upb1* (S8 Fig). This result indicates that *SHR* and *SCR* are involved in APP1 regulated the QC identity and root DSC differentiation.

### The regulation of root stem cell niche defining factors by ROS homeostasis

Since both increasing and decreasing ROS levels induced QC cell division and root DSC differentiation (Figs 5 and 6), the effect of expressing the *pPLT1*::*PLT1-YFP*, *pPLT2*::*PLT2-YFP*, *pSCR*::*SCR-GFP* and *pSHR*::*SHR-GFP* transgenes in the presence of either heightened or lowered levels of ROS was of interest. Under high ROS levels which were induced by H_2_O_2_ or MV treatments, the expression of both *pPLT1*::*PLT1-YFP* and *pPLT2*::*PLT2-YFP* was attenuated (S9A and S9B Fig), while that of both *pSCR*::*SCR-GFP* and *pSHR*::*SHR-GFP* was unaffected (S9C and S9D Fig). A low level of ROS, which was induced by catalase or DPI treatment, suppressed the *SCR* and *SHR* transgenes but not the *PLT1* or *PLT2* ones (S9 Fig). The suggestion is that the response to altered levels of ROS might be mediated by distinct signaling pathways.

## Discussion

### APP1 activity is essential for ROS homeostasis-dependent QC cell division and DSC maintenance

Given that in the *app1* mutant, the rate of cell division in the QC was higher than in the WT, and root DSC differentiation was more frequent (Fig 1), the conclusion is that APP1 has a crucial role in both processes. APP1 is one member of the P-loop NTP hydrolase family; these enzymes provide the energy required to generate conformational changes in many molecules [32]. That hydrolase activity of APP1 was confirmed *in vitro*, and more specifically, in the absence of APP1, the activity of the mitochondrial complex I was substantially reduced (Fig 3). Since the mitochondrion is one of the major producers of ROS in both mammalian cells and in plant cells [33,34], any disruption to mitochondrial complex I function is likely to alter cellular ROS status (Fig 4). UPB1 was recently reported to control root growth through the regulation of ROS gradient [24]. However, it seems that there was no reciprocal regulation between *APP1* and *UPB1*. In the *upb1* mutant, the expression of *APP1* is similar to the wild type, in the meanwhile, the *UPB1* expression was also not altered in *app1* (S8 Fig).The reduced ROS level in the *app1* mutant appeared to enhance cell division in the QC and the extent of root DSC differentiation, an effect which was reversed when the roots were exposed to either H_2_O_2_ or MV which induces the O^2-^ overproduction (Fig 5). However, besides influencing the hydrolysis of NTP, how APP1 affects the activity of mitochondria complex I remains challenging for future studies.

### An appropriate ROS level is important for the maintenance of stem cell identity

ROS not only function as passive defense signaling molecules, but also regulate cell survival and proliferation in response to both internal developmental signals and external environmental cues [35,36,37]. In mammals, ROS have been reported to play an important role in governing stem cell identity and controlling stem cell differentiation [38]. In contrast to the conventional idea of ROS as the “enemy of stem cells”, new evidences showed that the appropriate level of ROS is important for normal hematopoietic stem cell (HSC) function [39]. Using *in vivo* imaging, ROS was found to regulate the homing of HSCs to the bone marrow after transplantation, indicating that ROS plays a bilateral role in stem cell maintenance, and an appropriate level of ROS is required for normal hematopoietic stem cell function [40]. In *A*. *thaliana*, two of the main ROS molecules, O^2−^ and H_2_O_2_ distributed in dividing and expanding cells in the meristem and elongation zones, respectively, and overlap within the “transition zone” in root [24]. UPB1 was recently reported to regulate this ROS gradient through directly controlling the expression of a set of peroxidases and thus direct the transition between root cell proliferation and differentiation [24]. Disruption of UPB1 activity or overexpression of *UPB1* alters this ROS balance, leading to a delay or advance in the onset of differentiation and a longer root or shorter root respectively [24]. The transcription factor *UPB1* appears to regulate the ROS gradient by controlling the expression of genes encoding various peroxidases [24]. Here, the observation was that any disturbance (either an increase or a decrease) in the ROS level both induced cell division in the QC and enhanced the extent of root DSC differentiation. This result suggests that an optimal ROS level is important to maintain the QC and root DSC identity. ROS, as signaling molecules, has a conserved role to maintain stem cell identity. However, the role of ROS gradient in the maintenance of QC and root DSC remain to be elucidated. In addition, the mechanisms about how stem cells maintain appropriate levels of ROS are still waiting for further studies in the future.

### APP1 regulates QC cell division and root DSC identity without influencing auxin signaling root tip

The controlling ROS toxicity while enabling ROS such as H_2_O_2_ or O^2-^ to act as signaling molecules appears to a big challenge to plants. To achieve this fine regulation, the key role of plant hormones such as abscisic acid (ABA), salicylic acid (SA), Gibberellin (GA), ethylene and auxin have come into light [41]. Recently, auxin was found to inhibit the photorespiratory-dependent cell death in the *cat2* mutant, implying a role for auxin signalling in photorespiratory H_2_O_2_-dependent cell death [42]. In addition, another study from the same lab demonstrated that the perturbed mitochondria negatively affect the auxin signaling machinery [43]. Though mitochondria localized APP1 also affected the function of mitochondria and ROS homeostasis, auxin signaling in the *app1* mutant root tip was unaffected, which implied that APP1 might regulate QC cell division and root DSC identity without influencing auxin signaling. Consistent with this conclusion is the recent observation that the *UPB1*-controlled maintenance of root meristem identity is similarly independent of any auxin or cytokinin signaling [24].

### The participation of *SHR* and *SCR* in APP1 regulated the QC identity and root DSC differentiation

In animals, low ROS levels are critical for the maintenance of the self-renewal potential of stem cells, while high levels shut down self-renewal and drive stem cell differentiation. Certain ROS-responsive factors (p53, FoxOs, Nrf2, APE1/Ref-1 and HIF-1a) have been implicated as regulators of ROS-mediated stem cell self-renewal [44,45]. Although the importance of ROS for root meristem function is not in doubt, the mechanisms whereby it controls stem cell self-renewal remain largely unclear. The hormone abscisic acid interacts with ROS signaling by regulating the production of ROS in the plasma membrane and mitochondria [46,47]. The gene *ABO8* encodes a pentatricopeptide repeat protein which controls the splicing of *NAD4;* in the *abo8-1* mutant, ROS accumulates to a higher level than in the WT, and its root meristem is reduced in size due to the down-regulation of *PLT1* and *PLT2* [26]. Here, it was shown that exposure to either MV (which induces the production of superoxide) or H_2_O_2_ strongly down-regulated *PLT1*. At the same time, neither exposure to catalase or DPI (both of which reduced ROS levels) had any effect on the abundance of PLT1 and PLT2 protein accumulation (S9A and S9B Fig). Although ROS levels were lower in the *app1* mutant than in WT (Fig 4A–4D), the transcription of both *PLT1* and *PLT2* was not influenced in *app1* (S5A, S5B and S5D Fig); this can be taken to indicate that the effect on QC cell division and root DSC differentiation induced by a low ROS level operates via a pathway independent of both *PLT1* and *PLT2*. This was not the case for both *SHR* and *SCR*, which were clearly down-regulated in the *app1* mutant (Fig 7). Accordingly, both catalase and DPI treatment had a negative effect on the expression of both genes (S9C and S9D Fig). The evidence therefore supports a strong association between *APP1* and *SHR/SCR* dependent cell QC and root DSC identity. This study together with previous reports suggests that ROS acts as a binary switch controlling root development. There is an optimal ROS level, maintained through controlling the balance between ROS generation and ROS scavenger, to regulate DSCs identity in root. Either increasing or decreasing ROS levels will promote root DSC differentiation. The future challenge is to characterize new ROS responsive factors to address the molecular mechanism of ROS regulated root DSC identity.

## Materials and Methods

### Plant materials and growth conditions

*A*. *thaliana* ecotype Columbia-0 (Col-0) and transgenic lines expressing one of the constructs *pWOX5*::*GFP* [13], *QC184* [48], *pPLT1*::*PLT1-YFP* [49], *pPLT2*::*PLT2-YFP* [49], *pSHR*::*SHR-GFP* [50], *pSCR*::*SCR-GFP* [51], *DR5rev*::*GFP* [52], *pCYCB1;1*::*GUS* [53] and *Mito-cpYFP* [28] were employed, along with the *app1-1* (*Salk_110543*) and *app1-2* (*Salk_091643*) T-DNA mutants. Seeds were surface-sterilized with chlorine gas and held for three days at 4°C, plated onto Murashige and Skoog (MS) medium, then exposed to a 16 h photoperiod at a constant 20°C.

### TAIL-PCR analysis

Genomic DNA was extracted from leaves of WT Col-0 and the *app1-1* mutant using a CTAB-based method. The first PCR employed the T-DNA specific primer LBa1 along with a degenerate AD primer (sequences given in S1 Table), the second PCR combined LBb1.3 with AD primer and the third was primed with LBb1 and AD. Amplicons were separated by agarose gel electrophoresis and the *app1-1* mutant-specific and WT fragments were sequenced to identify the T-DNA insertion site. The primers used to TAIL-PCR analysis are provided in S1 Table.

### DNA constructs and plant transformation

The *APP1* (*At5g53540*) promoter was amplified from WT genomic DNA and inserted into either pDonr P4P1R or pDonr221 (Invitrogen, Carlsbad, CA, USA) according to the manufacturer's protocol. *APP1* cDNA was inserted into pDONR221. The *pAPP1*::*GUS-GFP* construct was generated using Gateway technology (www.thermofisher.com/gateway.html) [54] by inserting the *APP1* promoter into the binary vector pKGWFS7. The *pAPP1*::*GFP-APP1* construct was similarly generated [54] by inserting the *APP1* promoter and cDNA into the binary vector pB7m34GW. The *p35S*::*GFP-APP1* construct was constructed by introducing the *APP1* cDNA into the binary vector pK7WGF2.0. Each of the transgenes was first transformed into *Agrobacterium tumefaciens* GV3101 and from thence into a WT *A*. *thaliana* plant using the floral dip method. The primers used to generate constructs are provided in S1 Table.

### GUS, Lugol and EdU staining

Staining of seedling roots for GUS activity was carried out by incubation at 37°C in 0.05M NaPO_4_ buffer (pH 7.0), 5mM K_3_Fe(CN)_6_, 5mM K_4_ Fe(CN)_6_ and 2mM X-glucuronide. Once the color had developed, the material was passed through an ethanol series (70%, 50% and 20%) before mounting in 70% chloral hydrate in 10% v/v glycerol. Detection of starch granules in the root tip was achieved by steeping seedlings in Lugol’s solution for 1–2 min, after which seedlings were mounted in chloral hydrate as above. EdU staining was performed following the protocol supplied with the Click-iT EdU Alexa Fluor 647 Imaging kit (Invitrogen). Five-day-old seedlings grown on medium were cultured with 10μM EdU for 12h before EdU incorporation in the QC was examined. After incubation, remove the media and add 3.7% formaldehyde in PBS buffer, incubate for 15 minutes at room temperature, then wash the seedlings with 3% BSA in PBS. Add 0.5% Triton X-100 in PBS buffer, incubate at room temperature for 20 minutes. After washing, add 0.5 mL of Click-iT reaction cocktail, incubate for 30 minutes, protected from light. Observations were made using confocal microscopy.

### Subcellular localization of APP1

*pAPP1*::*APP1-GFP* seedlings were stained with the Mito-tracker Red (Invitrogen) and examined by laser-scanning confocal microscopy. Green (GFP) images were obtained using an excitation wave length of 488 nm and a capture wavelength of 525 nm. Red (Mito-Tracker stained) images were obtained using an excitation wave length of 543 nm and a capture wavelength of 615 nm. Isolation of mitochondria proteins from *pAPP1*::*APP1-GFP* seedlings through Mitochondrial protein extraction kit (NanJing JianCheng Bioengineering Institute, G008). Anti-GFP antibody (Abcam, ab290) and anti-COX IV antibody (Abcam, ab16056) were used for western blotting detection.

### RNA analysis

Seedlings were grown on MS medium for five days, after which the distal 5 mm was harvested. Total RNA was isolated using a RNeasy Mini Kit (Qiagen) and the first cDNA strand synthesized from a 2 μg aliquot using a Transcriptor First Strand cDNA Synthesis kit (Roche), following the manufacturer’s protocol. Quantitative real-time PCRs were based on the CFX Connect Real-Time System (Bio-Rad) and the Fast Start Universal SYBR Green Master mix (Roche). Three biological replicates were included, each of which was represented by three technical replicates. The *AtACTIN2* sequence was used as the reference. The primers used to quantify the gene expression levels are provided in S1 Table.

### Phenotypic analysis, microscopy, statistics

Seedlings grown on MS were scanned and root lengths were determined from the digital images using Image J software. Confocal imaging was obtained using an LSM-700 laser-scanning confocal microscope (Zeiss). The statistical significance of differences between means was determined using the Student’s t-test.

### Isolation of His fusion APP1 proteins from *E*. *coli*

The open reading frame of *APP1* fused with six His at the C terminus, was cloned into a pET vector and expressed in *E*.*coli*. Pre-cultures were grown overnight at 37°C in Luria-Bertani medium. Samples (10 mL) of these pre-cultures were used to inoculate 100 mL of fresh Luria-Bertani medium at 28°C. After growing the culture to a density of OD 600 = 0.8 to 1.0, APP1 protein expression was induced with 0.5 mM IPTG for 4 h at 28°C. The isolation of His fusion APP1 protein was done by Ni sepharose from GE healthcare.

### Histochemical assays

The H2-DCFDA (Invitrogen) staining assay was performed as described elsewhere [24], and the resulting fluorescence detected by confocal microscopy, using an excitation wavelength of 488 nm and an emission wavelength of 525 nm. The presence of H_2_O_2_ was detected in sample roots by steeping in 0.1 mg/mL DAB (Sigma) in 50 mM Tris-HCl (pH 5.0. NBT staining was used to detect O^2-^ using a method modified from that described by [55]. Four-day-old seedlings were incubated in 2 mM NBT (Sigma) in 20 mM K phosphate / 0.1 M NaCl at pH 6.1 for 15 min, and then transferred to distilled water. To assay for ATP hydrolase activity, a reaction mixture comprising 50 mM Tris-HCl (pH 7.5), 50 mM NaCl, 0.1 mM EDTA, 1.5 mM DTT, 10 mM MgCl_2_, 10% v/v glycerol, 2mM ATP and various concentrations of the test proteins was held at room temperature for 30 minutes. ATP hydrolase activity was showed in the form of pmol ATP hydrolyzed per pmol protein per min, based on the Pi ColorLock Gold Phosphate Detection System (Innova Bioscience). To assay for mitochondrial NADH dehydrogenase activity, mitochondria were isolated from WT and the *app1-1* mutant seedlings, according to the mitochondrial isolation kit (GENMED). Enzyme activity was assessed spectrophotometrically at 340 nm. To assay for measurement of H_2_O_2_ content, 5-day-old seedlings of WT and the *app1* mutants were grinded with H_2_O_2_ extraction buffer (Beyotime Biotechnology) at 4°C, the content was assessed spectrophotometrically at 560 nm.

### Western blotting

Total plant proteins of transgenic lines such as *pSCR*::*SCR-GFP pSCR*::*SCR-GFP*/*app1-1*, *pSCR*::*SCR-GFP/app1-2*, *pSHR*::*SHR-GFP*, *pSHR*::*SHR-GFP*/*app1-1* and *pSHR*::*SHR-GFP*/*app1-2*, were prepared by grinding plant tissue in liquid nitrogen and then extracting in 50mM Tris-HCl (pH 8.0), 150mM NaCl, 1% v/v Nonidet P-40, 0.5% w/v sodium deoxycholate, 0.1% w/v SDS and 1 mM phenylmethylsulfonyl fluoride. After centrifugation (14,000g, 15 min, 4°C), the supernatant was collected and then subjected to SDS–PAGE. Following the electrophoresis, the separated proteins were transferred onto a polyvinylidene difluoride membrane, and detected by probing first with a 1:5,000 dilution of primary antibody of GFP (Abcam), followed by a 1:10,000 dilution of horseradish peroxidase-conjugated goat anti-rabbit IgG (Beijing Dingguo Changsheng Biotechnology). The signal was detected using a SuperSignal Western Femto Maximum Sensitivity Substrate kit (Thermo Scientific), following the manufacturer’s instructions.

## Supporting Information

S1 Fig The confirmation of *app1* mutants and APP1-OE lines. (A)The sites of T-DNA insertion in the two app1 mutants. Boxes and lines represent, respectively, exons and introns. (B) *app1-1 app1-2* F1 hybrid showed the high frequency of QC division and DSC differentiation. (C) Quantitative RT-PCR analysis suggests the suppression of *APP1* in the mutants. (D) pAPP1:APP1-GFP complemented the QC division and DSC differentiation defects of *app1-1* and *app1-2* mutants. (E) APP1 transcript abundance in WT and the *APP1* over-expression lines. The data are given in the form of the mean with an associated SD (n = 3); *: P<0.05, **: P<0.01. Student’s t test.
(TIFF)

S2 Fig Phenotypes of the *app1* mutants. (A) The length of the primary roots measured eight days after germination. (B) Eight days old seedlings of WT, *app1* mutants and *APP1 OE* grown on MS medium. (C) The length of the meristem zone measured five days after germination. (D) Quantification of GFP fluorescence of *WOX5* in Col and *app1* mutants. The data are given in the form of the mean with an associated SD (*n* = 3).
(TIFF)

S3 Fig The detection of ROS levels after treatment with MV, DPI, H_2_O_2_ and catalase. (A) Confocal images of Mito-cpYFP after treatment with MV and DPI. (B) Confocal images of H2-DCFDA after treatment with H_2_O_2_ and catalase. Bar: 50 μm.
(TIFF)

S4 Fig The QC-specific expression of *APP1* increases the local rate of cell division and the extent of root DSC differentiation. (A) Quantification of QC cell division (black bar) and root DSC differentiation (gray bar). The presence of the pWOX5::APP1 transgene in a WT background increases QC cell division by around 30% and root DSC differentiation by around 15%. In an app1 background, the same transgene partially negates the mutation's effect on both QC cell division and root DSC identity.
(TIFF)

S5 Fig Expression profiling in both a wt and an *app1* background of transgenic plants harboring (A) *pPLT1*::*PLT1-YFP*, (B) *pPLT2*::*PLT2-YFP* and (C) *pCYCB1;1*::*GUS*. Bar: 50 μm. (D)The relative expression level of *PLT1*, *PLT2*, *CYCB1* and *WOX5* in WT and *app1* mutants. The data are given in the form of the mean with an associated SD (n = 3).
(TIFF)

S6 Fig The relative expression level of *PLT1* and *PLT2* in WT and *APP1-OE* lines. (A). The data are given in the form of the mean with an associated SD (n = 3).
(TIFF)

S7 Fig The auxin signaling response of the *app1* mutant is indistinguishable from that of the WT. (A) The figure illustrates the expression in the root of the *DR5rev*::*GFP* transgene in both a WT and *app1* background. Bar: 50 μm.
(TIFF)

S8 Fig The relative expression level of *UPB1* in WT and *app1* mutants (A) and the relative expression level of *APP1* in WT and *upb1* mutant (B). The data are given in the form of the mean with an associated SD (n = 3).
(TIFF)

S9 Fig The effect of ROS level changes on the expression of *PLT1*, *PLT2*, *SCR* and *SHR*. The expression of the transgenes (A) *pPLT1*::*PLT1-YFP*, (B) *pPLT2*::*PLT2-YFP*, (C) *pSCR*::*SCR-GFP* and (D) *pSHR*::*SHR-GFP* in the presence or absence of H_2_O_2_, catalase, MV or DPI. (E) Quantification of GFP fluorescence shown in (A, B, C, D). (F) The expression level of *PLTs* in *APP1-OE* lines. Bar: 50 μm. **: P<0.01. Student’s t test.
(TIFF)

S1 Table Primers used in this study. (DOC)
