# Supplementary figures and images for: A P-Loop NTPase Regulates Quiescent Center Cell Division and Distal Stem Cell Identity through the Regulation of ROS Homeostasis in Arabidopsis Root
Source: PLoS Genet. 2016 Sep 1;12(9):e1006175. doi: 10.1371/journal.pgen.1006175 (PMC5008728; doi:10.1371/journal.pgen.1006175)

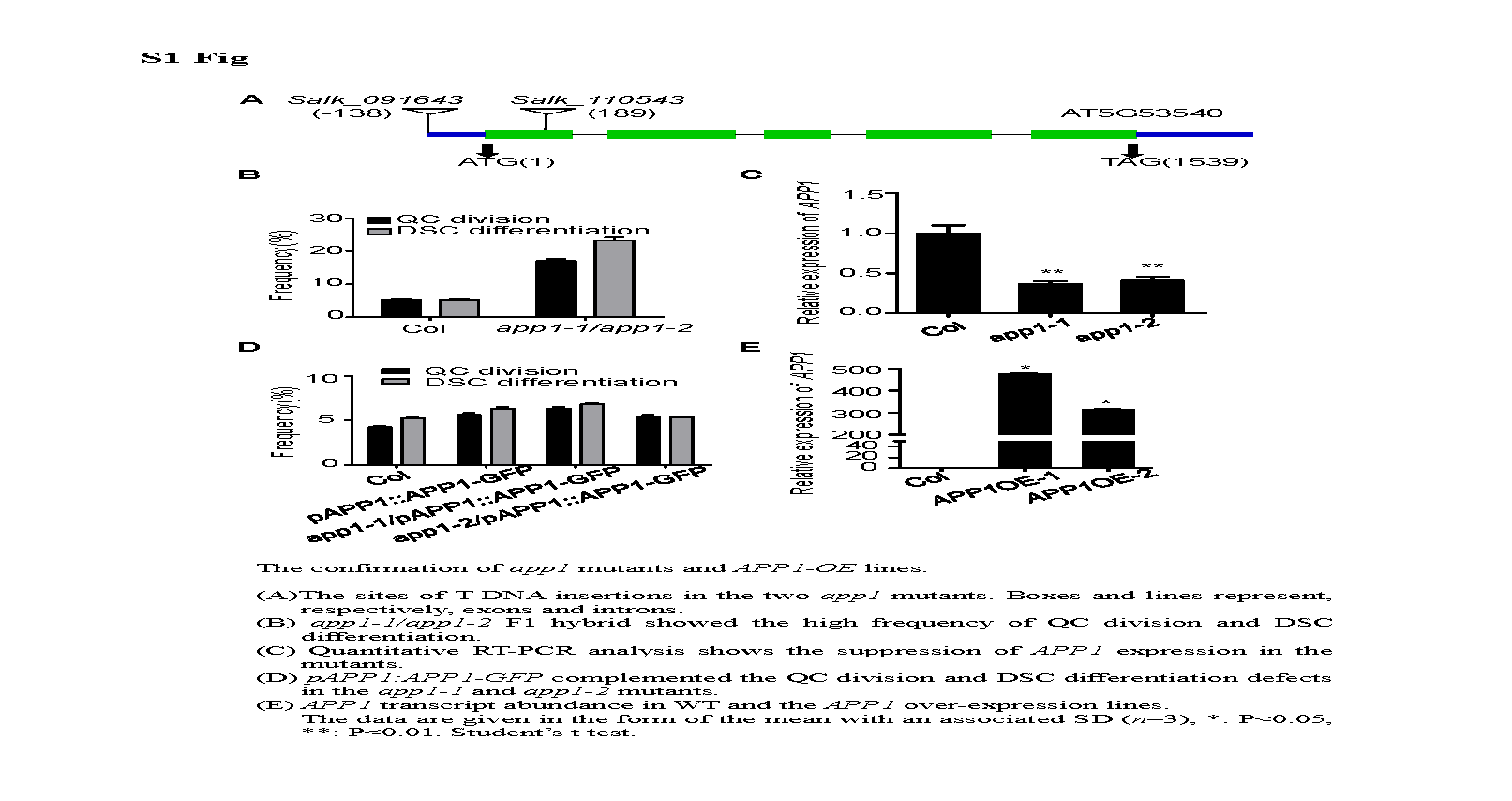

Supplement: S1 Fig — (A)The sites of T-DNA insertion in the two app1 mutants. Boxes and lines represent, respectively, exons and introns. (B) app1-1 app1-2 F1 hybrid showed the high frequency of QC division and DSC differentiation. (C) Quantitative RT-PCR analysis suggests the suppression of APP1 in the mutants. (D) pAPP1:APP1-GFP complemented the QC division and DSC differentiation defects of app1-1 and app1-2 mutants. (E) APP1 transcript abundance in WT and the APP1 over-expression lines. The data are given in the form of the mean with an associated SD (n = 3); *: P<0.05, **: P<0.01. Student’s t test. (TIFF) [file pgen.1006175.s001.tiff]

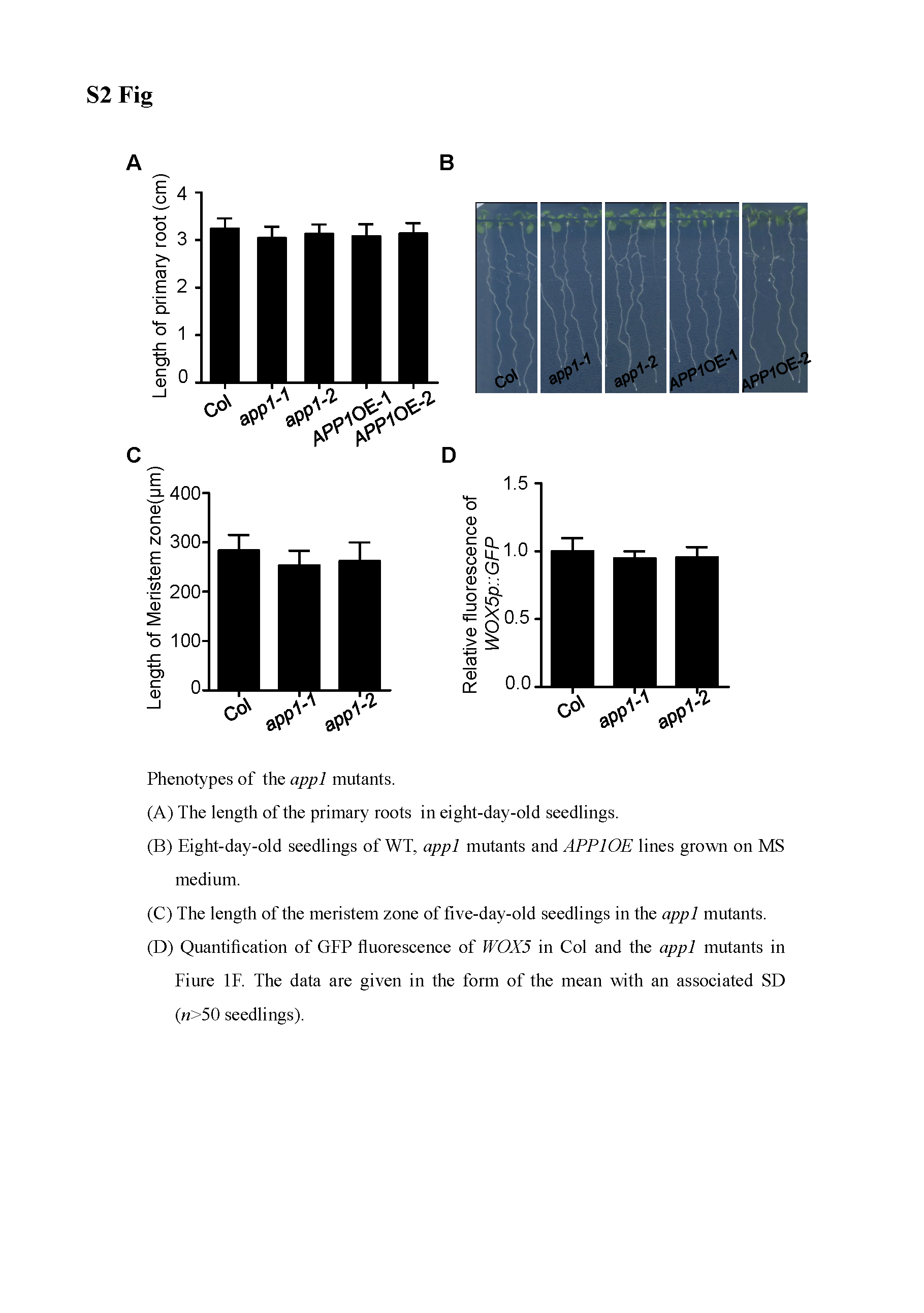

Supplement: S2 Fig — (A) The length of the primary roots measured eight days after germination. (B) Eight days old seedlings of WT, app1 mutants and APP1 OE grown on MS medium. (C) The length of the meristem zone measured five days after germination. (D) Quantification of GFP fluorescence of WOX5 in Col and app1 mutants. The data are given in the form of the mean with an associated SD (n = 3). (TIFF) [file pgen.1006175.s002.tiff]

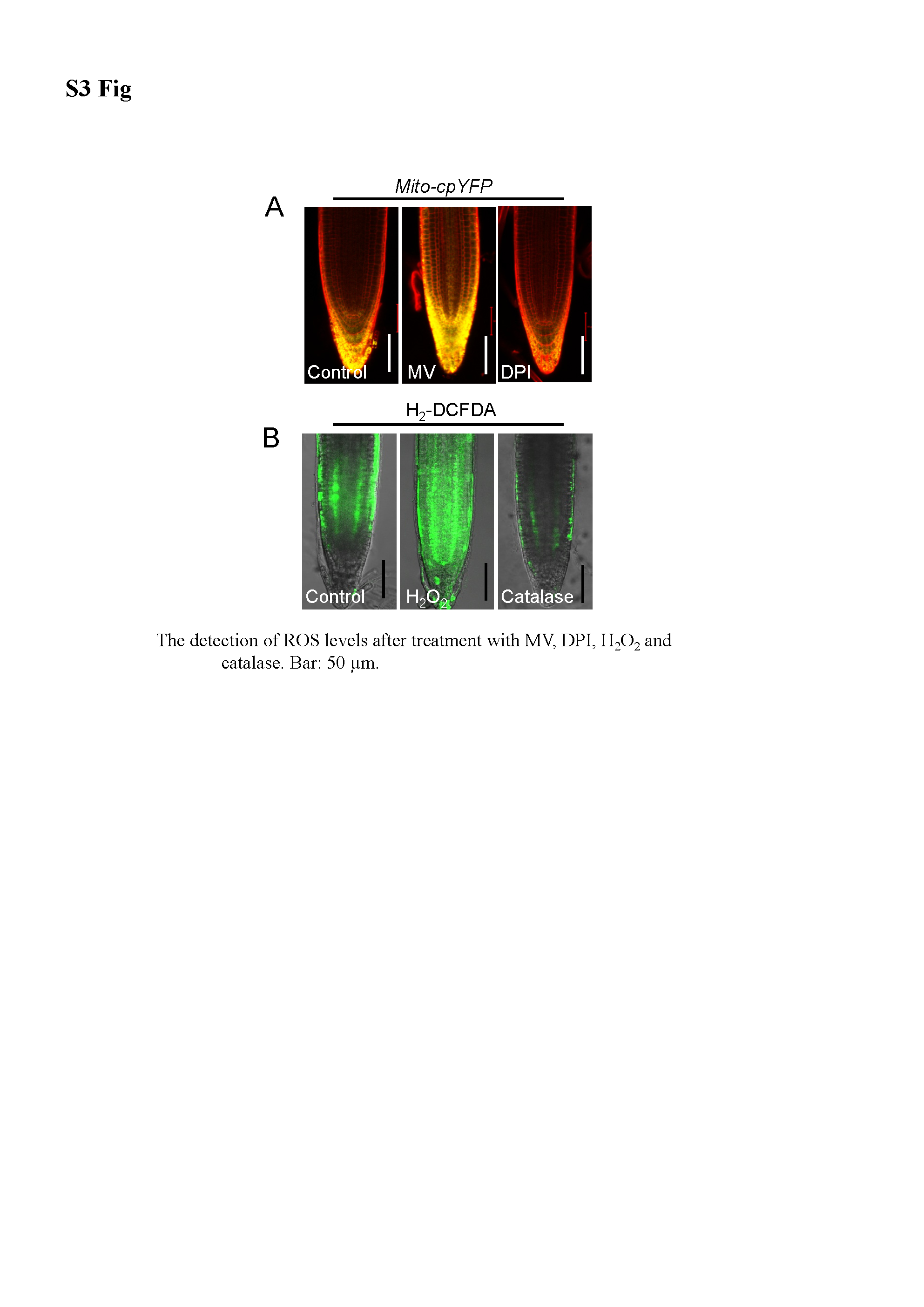

Supplement: S3 Fig — (A) Confocal images of Mito-cpYFP after treatment with MV and DPI. (B) Confocal images of H2-DCFDA after treatment with H2O2 and catalase. Bar: 50 μm. (TIFF) [file pgen.1006175.s003.tiff]

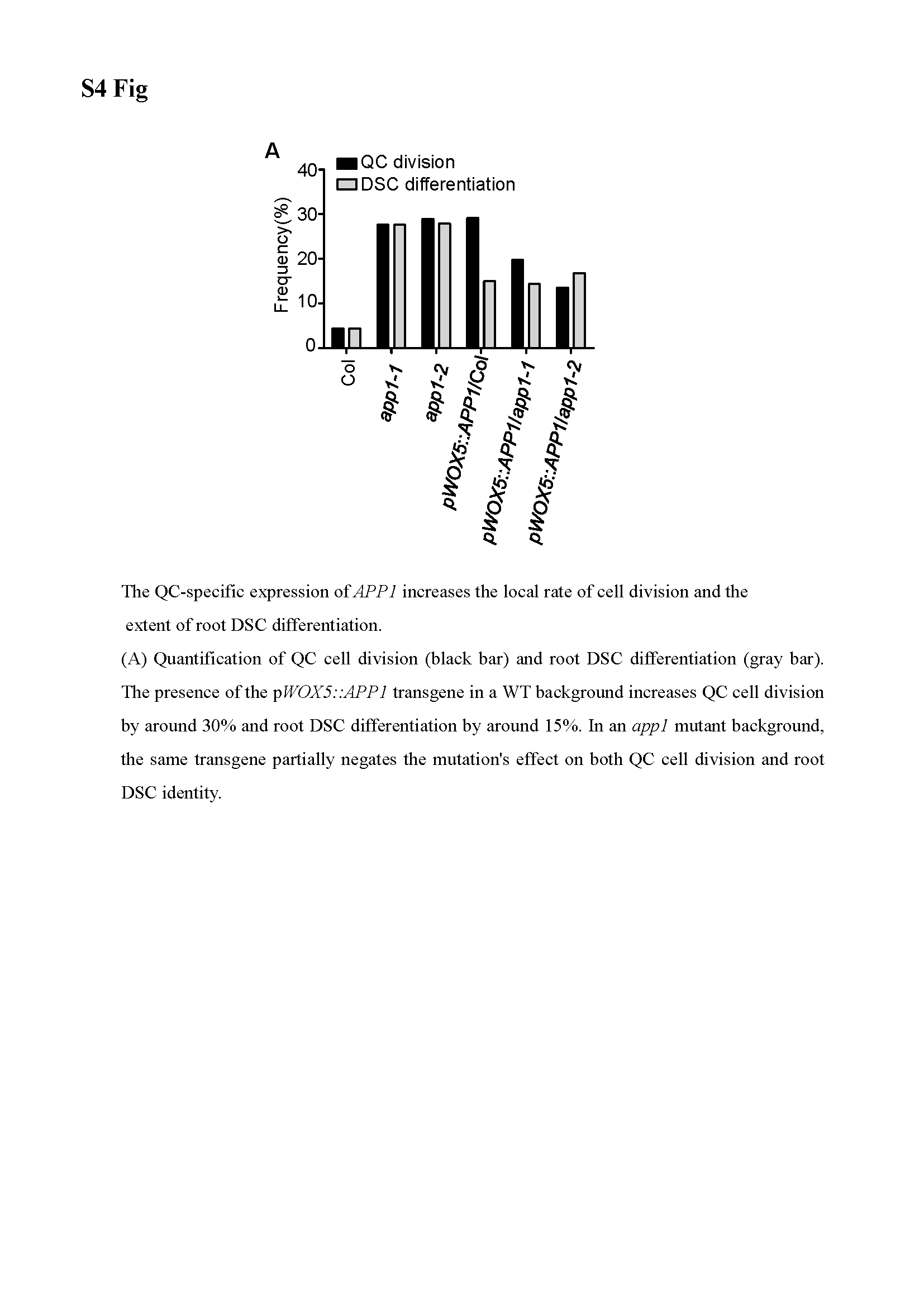

Supplement: S4 Fig — (A) Quantification of QC cell division (black bar) and root DSC differentiation (gray bar). The presence of the pWOX5::APP1 transgene in a WT background increases QC cell division by around 30% and root DSC differentiation by around 15%. In an app1 background, the same transgene partially negates the mutation's effect on both QC cell division and root DSC identity. (TIFF) [file pgen.1006175.s004.tiff]

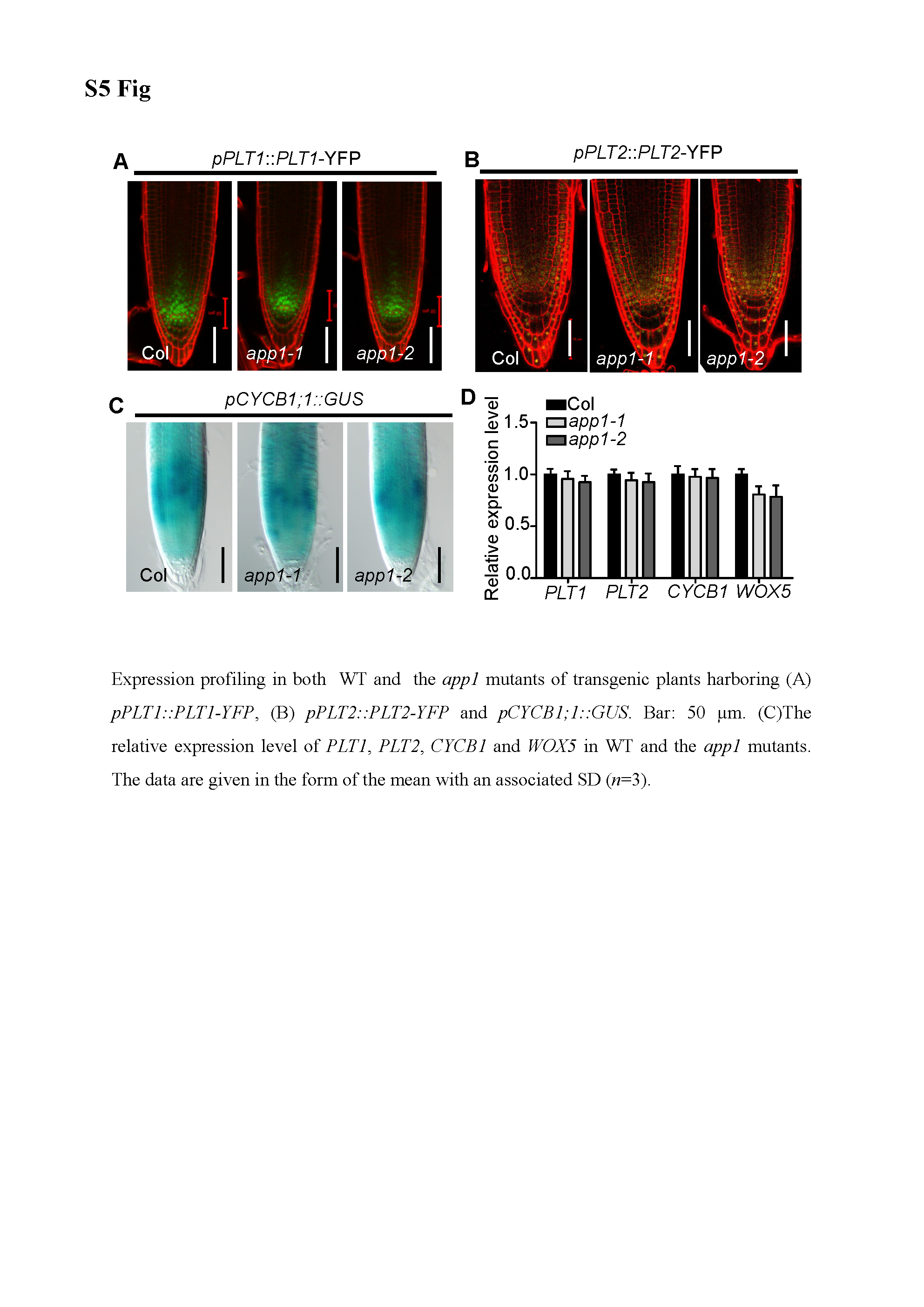

Supplement: S5 Fig — Expression profiling in both a wt and an app1 background of transgenic plants harboring (A) pPLT1::PLT1-YFP, (B) pPLT2::PLT2-YFP and (C) pCYCB1;1::GUS. Bar: 50 μm. (D)The relative expression level of PLT1, PLT2, CYCB1 and WOX5 in WT and app1 mutants. The data are given in the form of the mean with an associated SD (n = 3). (TIFF) [file pgen.1006175.s005.tiff]

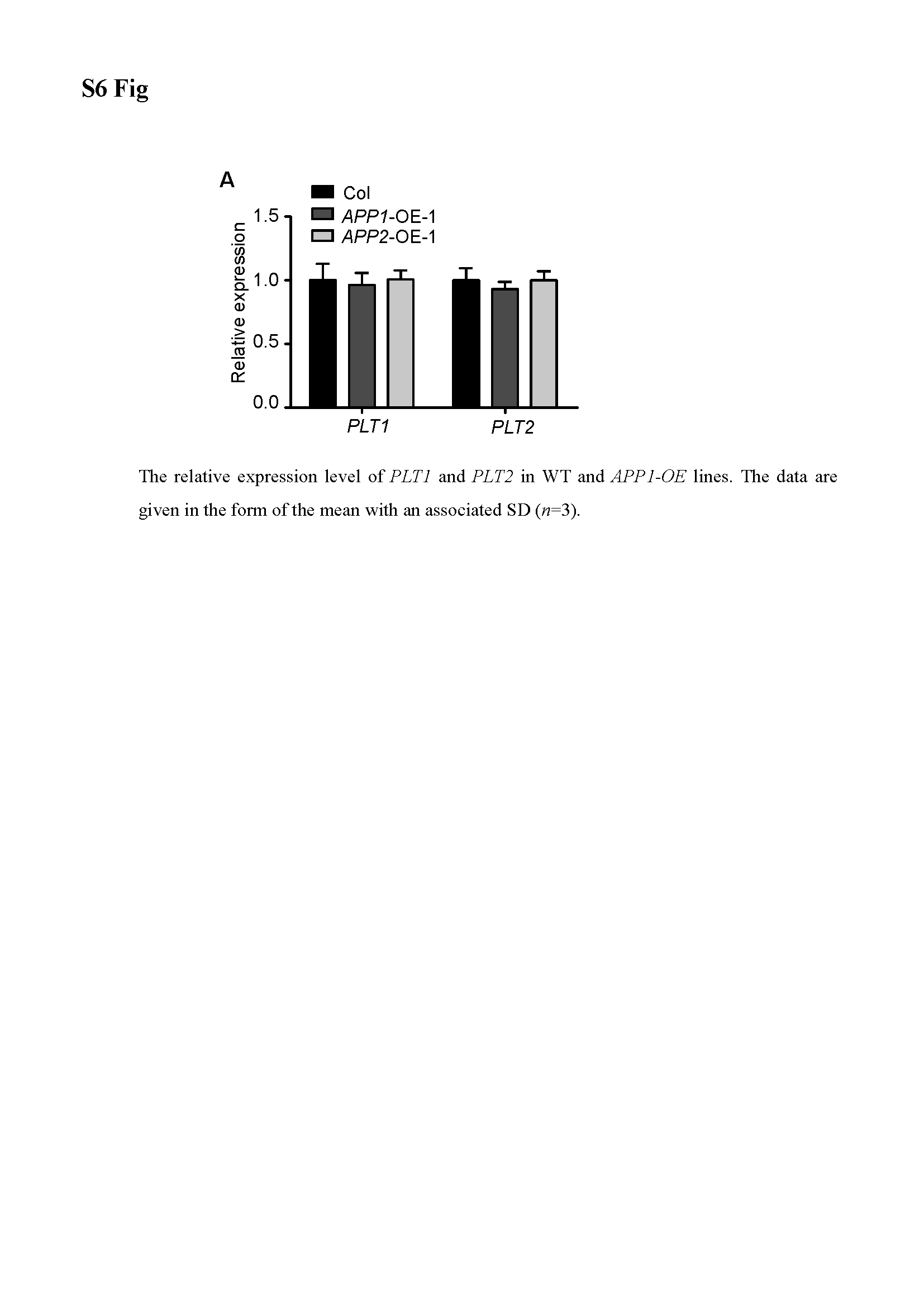

Supplement: S6 Fig — (A). The data are given in the form of the mean with an associated SD (n = 3). (TIFF) [file pgen.1006175.s006.tiff]

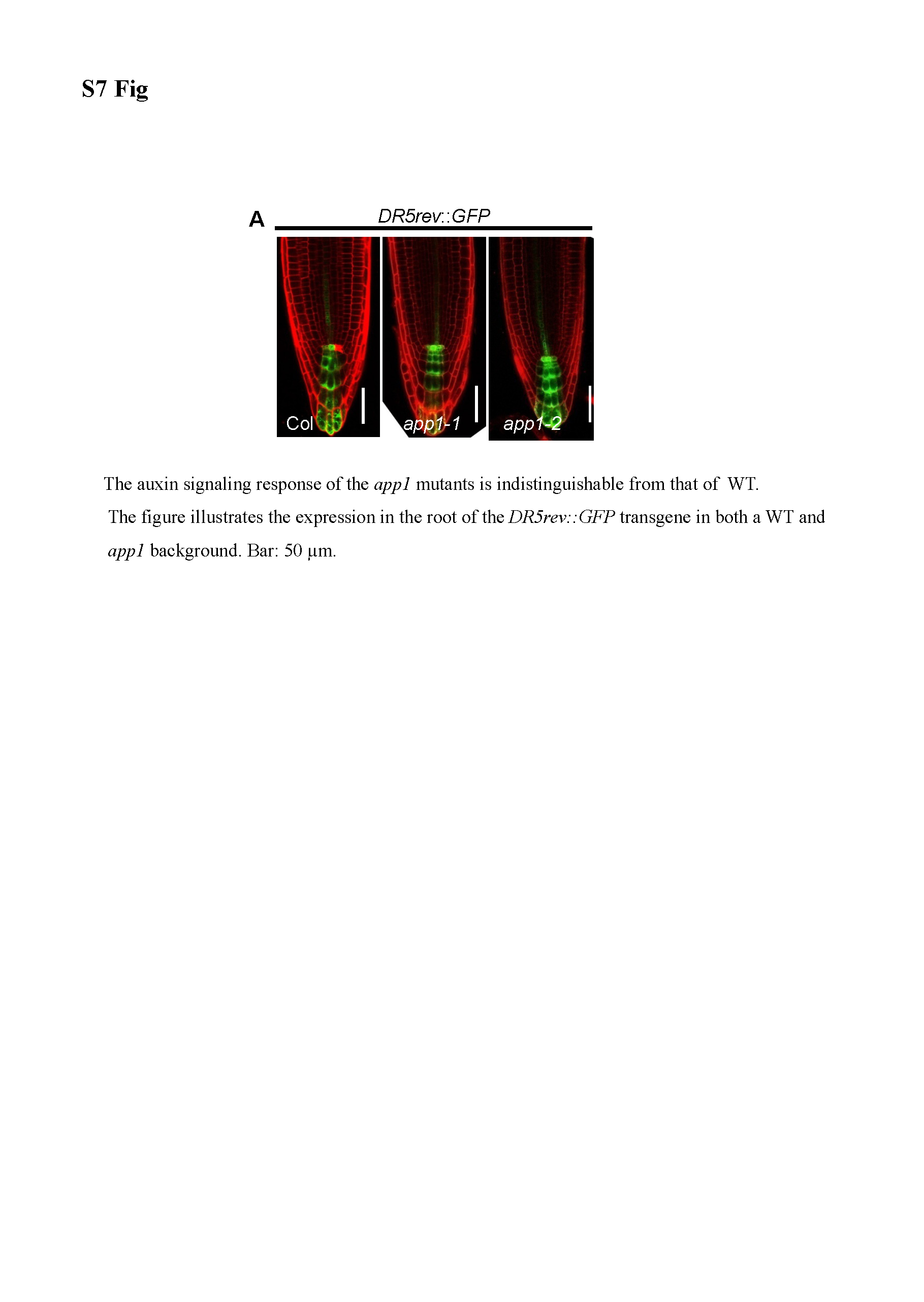

Supplement: S7 Fig — (A) The figure illustrates the expression in the root of the DR5rev::GFP transgene in both a WT and app1 background. Bar: 50 μm. (TIFF) [file pgen.1006175.s007.tiff]

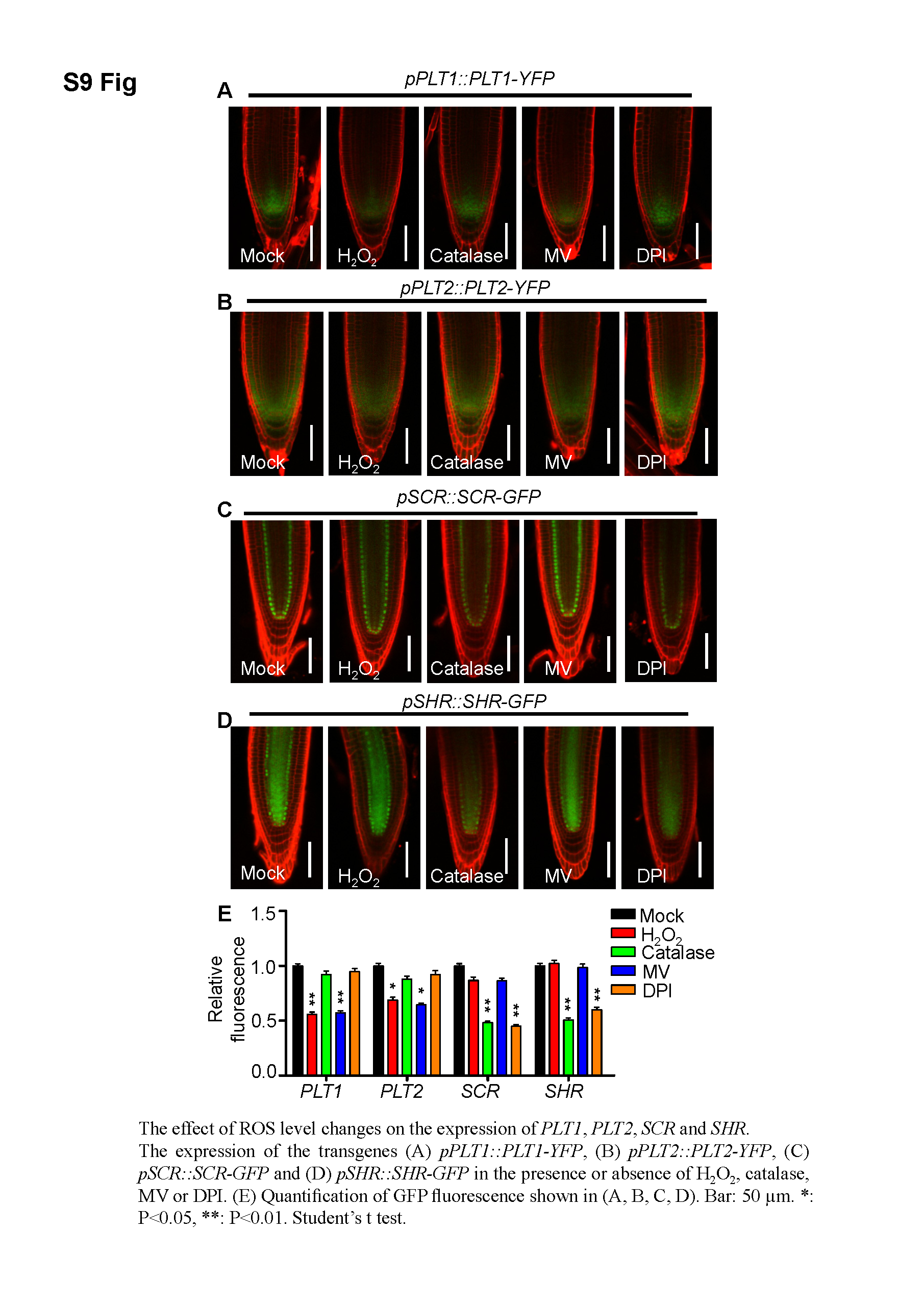

Supplement: S9 Fig — The expression of the transgenes (A) pPLT1::PLT1-YFP, (B) pPLT2::PLT2-YFP, (C) pSCR::SCR-GFP and (D) pSHR::SHR-GFP in the presence or absence of H2O2, catalase, MV or DPI. (E) Quantification of GFP fluorescence shown in (A, B, C, D). (F) The expression level of PLTs in APP1-OE lines. Bar: 50 μm. **: P<0.01. Student’s t test. (TIFF) [file pgen.1006175.s009.tiff]
