# Supplementary material for: A P-Loop NTPase Regulates Quiescent Center Cell Division and Distal Stem Cell Identity through the Regulation of ROS Homeostasis in Arabidopsis Root
Source: PLoS Genet. 2016 Sep 1;12(9):e1006175. doi: 10.1371/journal.pgen.1006175 (PMC5008728; doi:10.1371/journal.pgen.1006175)
Supplement: S1 Table — (DOC) [file pgen.1006175.s010.doc]

| Primer | Sequence (5'-3') | Experiments |
| --- | --- | --- |
| APP1CF | GGGGACAAGTTTGTACAAAAAAGCAGGCTGCATGAAGAGATCTTCAGGGAGAAA | 35S:GFP::APP1 |
| APP1CR1 | GGGGACCACTTTGTACAAGAAAGCTGGGTCTTCCGAATCTCCTTCTGAAGGA | 35S:GFP::APP1 |
| APP1PF | GGGGACAAGTTTGTACAAAAAAGCAGGCTGCCAACCTATTTCAATCGGCCAGA | APP1p:: GFP/GUS |
| APP1PR | GGGGACCACTTTGTACAAGAAAGCTGGGTCCATTTCCGAATCTCCTTCTGAA | APP1p:: GFP/GUS |
| APP1PF1 | GGGGACAACTTTGTATAGAAAAGTTGGCCAACCTATTTCAATCGGCCAGA | APP1p::APP1:: GFP |
| APP1PR1 | GGGGACTGCTTTTTTGTACAAACTTGCCATTTCCGAATCTCCTTCTGAA | APP1p::APP1:: GFP |
| APP1RTF | CGAGGATGTGATTGCGTGTGATG | qRT-PCR |
| APP1RTR | GAGGACCCAGTAACTTCCCATAAG | qRT-PCR |
| AD1 | NGTCGASWGANAWGAA | Tail-PCR |
| AD2 | TGWGNAGSANCASAGA | Tail-PCR |
| AD3 | AGWGNAGWANCAWAGG | Tail-PCR |
| AD4 | STTGNTASTNCTNTGC | Tail-PCR |
| AD5 | NTCGASTWTSGWGTT | Tail-PCR |
| AD6 | WGTGNAGWANCANAGA | Tail-PCR |
| SHR-F | TGGTCGAGGAGGATGAGGAATAG | qRT-PCR |
| SHR-R | ACACTGTACCATCGACCAAACACC | qRT-PCR |
| SCR-F | TAGCGGTTGGAGGACCATCG | qRT-PCR |
| SCR-R | CGCTTGTGTAGCTGCATTTCC | qRT-PCR |
| CYCB1;1-F | CCGGAACTGAATCTGCTTAGG | qRT-PCR |
| CYCB1;1-R | GCGACTCATTAGACTTGTTCA | qRT-PCR |
| ACT2-F | TTGACTACGAGCAGGAGATGG | qRT-PCR |
| ACT2-R | ACAAACGAGGGCTGGAACAAG | qRT-PCR |
| PLT1-F | TCGCCGGAAACAAAGAC | qRT-PCR |
| PLT1-R | CCGATGGGAAGAGTGCTAC | qRT-PCR |
| PLT2-F | ACTCTTCTTTGCCGCCTCACAT | qRT-PCR |
| PLT2-R | CCCCCGATTTGCTCACTCC | qRT-PCR |
| WOX5-F | GATTGTCAAGAGGAAGAGAAGGTGA | qRT-PCR |
| WOX5-R | AGCTTAATCGAAGATCTAATGGCG | qRT-PCR |
| UPB1-F1 | GTTATGCAGACAATGGTTCAGG | qRT-PCR |
| UPB1-R1 | AGCAACACAAGACAATCACACG | qRT-PCR |
| PER11F | AGGATGTGATGGATCGGTGTT | qRT-PCR |
| PER11R | GAGTTGGAAGGTTTGTTGTGG | qRT-PCR |
| PER55F | TGTTCTTTCACGACTGCTTCGTC | qRT-PCR |
| PER55R | AGCTCCACCTTAAACTCTGGCCC | qRT-PCR |
